# Anticancer properties of curcumin-treated *Lactobacillus plantarum* against the HT-29 colorectal adenocarcinoma cells

**DOI:** 10.1038/s41598-023-29462-7

**Published:** 2023-02-17

**Authors:** Faranak Gholipour, Mohammad Amini, Behzad Baradaran, Ahad Mokhtarzadeh, Morteza Eskandani

**Affiliations:** 1 Department of Biological Science, Faculty of Basic Science, Higher Education Institute of Rab-Rashid, Tabriz, Iran; 2 Immunology Research Center, Tabriz University of Medical Sciences, Tabriz, Iran; 3 Research Center for Pharmaceutical Nanotechnology (RCPN), Biomedicine Institute, Tabriz University of Medical Sciences, Tabriz, Iran

**Keywords:** Cancer therapy, Microbiome, Symbiosis

## Abstract

Probiotic bacteria with functions of importance to the health and well-being of the host exhibit various medicinal properties including anti-proliferative properties against cancer cells. There are observations demonstrating probiotic bacteria and their metabolomics can be different in various populations with different eating habits. Here, *Lactobacillus plantarum* was treated with curcumin (the major compound of turmeric), and its resistance to the curcumin was determined. After then the cell-free supernatants of untreated bacteria (CFS) and bacteria treated with curcumin (cur-CFS) were isolated and their anti-proliferative properties against HT-29 colon cancer cells were compared. The ability of *L. plantarum* treated with curcumin to combat a variety of pathogenic bacterial species and its ability to survive in acidic conditions were evidence that the probiotic properties of the bacterium were unaffected by the curcumin treatment. *L. plantarum* treated with curcumin and intact *L. plantarum* were both able to live in acidic conditions, according to the results of the resistance to low pH test. The MTT result showed that CFS and cur-CFS dose-dependently decreased the growth of HT29 cells with a half-maximal inhibitory concentration of 181.7 and 116.3 µL/mL at 48 h, respectively. Morphological alteration of DAPI-stained cells also exhibited significant fragmentation in the chromatin within the nucleus of cur-CFS-treated cells compared to CFS-treated HT29 cells. Moreover, flow cytometry analyses of apoptosis and cell cycle confirmed DAPI staining and MTT assay results and stipulated the increased occurrence of programmed cell death (apoptosis) in cur-CFS-treated cells (~ 57.65%) compared to CFS-treated cells (~ 47%). These results were more confirmed with qPCR and exhibited the upregulation of *Caspase* 9–3 and *BAX* genes, and downregulation of the *BCL-2* gene in cur-CFS- and CFS-treated cells. In conclusion, turmeric spice and curcumin may affect the metabolomics of probiotics in intestinal flora which could subsequently influence their anticancer properties.

## Introduction

Despite significant advances in diagnosis and treatment, the prevalence of various cancers remains extremely high^1^. According to the NCI (National Cancer Institute), the number of new cases and deaths from colorectal cancer are expected to be 51,030 and 52,580 by the end of 2022, respectively. Nutrition appears to play a critical role in cancer prevention, initiation, and progression, in addition to various effective treatment modalities^2^. The digestive tract is a complex ecosystem teeming with both beneficial and harmful microorganisms^3^. The Lactobacillus bacteria (LAB) genus is one of the most common genera of probiotics in the small and large intestines, and it may appear in the colon as a result of consuming probiotic-containing meals^4^. Certain LABs (e.g.,* Lactobacillus casei, Lactobacillus rhamnosus,* and* Lactobacillus acidophilus*) control oncomarkers in human colon cancer and carcinogen-induced colon malignancies in experimental animal models^5^. LAB may also interact directly with tumor cells, preventing their proliferation^6^. Probiotic bacteria may have anticancer effects through a variety of mechanisms, including carcinogen elimination, alteration of colon physicochemical conditions, intestinal microflora metabolic activities, production of anti-mutagenic or anti-tumorigenic compounds, and stimulation of the immune system^7^. In clinical trials, prebiotics have been shown to increase the magnitude and metabolic activity of the LAB in the intestinal and colorectal systems of animals^8^. Food additives, on the other hand, are expected to alter the composition of the microbiome (particularly LAB), its secondary metabolites, and metabolic pathways^9^. Curcumin is a polyphenol derived from the spice and herbal remedies turmeric^10^. When taken orally or topically, it has a number of anti-inflammatory and anticancer properties. At both neutral and acidic pH levels, curcumin possesses potent antioxidant properties. It also influences cell signaling, enzyme activity, immunomodulation, angiogenesis, and cell–cell adhesion^11^. Curcumin’s ability to modulate gene transcription and induce apoptosis in preclinical models suggests that it may be especially useful for cancer chemoprevention and chemotherapy in humans^12^. Although curcumin’s low systemic bioavailability in oral administration may limit access to sufficient concentrations for pharmacological effects in certain tissues, evidence of biologically active levels in both animals and humans has been demonstrated^13^. Oral curcumin has already been studied extensively, and it appears to have the potential to help people with invasive or pre-invasive gastrointestinal tract cancers, particularly those of the colon and rectum^14^. Antioxidants may increase the effectiveness of the LAB as probiotics in the intestines, making them more effective against cancer^15^. The goal of this study was to see how curcumin affected the anticancer activity of probiotics. In this study, *L. plantarum* was used as the probiotic bacterium. The inhibitory properties of bacterial metabolites produced by *L. plantarum* treated with curcumin were then compared to those of metabolites produced by untreated *L. plantarum* as the control group. The cytotoxicity of secreted metabolites on the HT-29 human colorectal adenocarcinoma cell line was assessed using 3-(4,5-dimethylthiazol-2-yl)-2,5-diphenyltetrazolium bromide tetrazolium (MTT) assay, 4′,6 diamidine-2′-phenylindole dihydrochloride (DAPI) staining, flow cytometry analysis of apoptosis/necrosis, and DNA fragmentation. Finally, quantitative real-time PCR was used to examine gene expression changes that contribute to cell survival and apoptosis in order to better understand the mechanisms of action of bioactive metabolites of bacteria against cancer cells.

## Results

### Preparation of cell-free supernatants (CFS)

**Figure 1:**
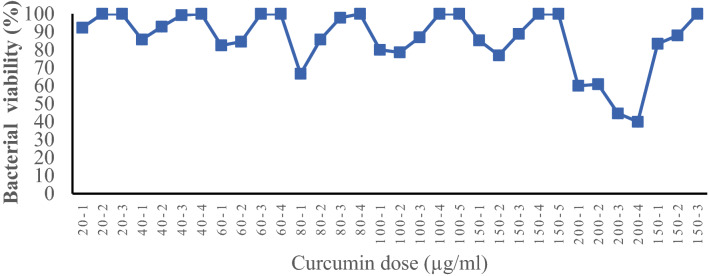
Adaptation of *Lactobacillus plantarum* to curcumin food additive.

The CFS of curcumin-treated *L. plantarum* was utilized to compare its impact with the CSF of intact *L. plantarum* against HT-29 colon cancer cells. Repetitive treatment with various concentrations of curcumin (20–150 µg/mL) was applied to adapt *L. plantarum* to the highest doses of curcumin (150 µg/mL). This was done through adaptive/repetitive bacterial cultures in the presence of gradient curcumin concentrations, followed by the collection of the resultant CFS for further investigation. The bacterial culture procedure was repeated for each curcumin concentration until the bacterial populations reached the desired CFU. In the presence of curcumin, the number of bacterial colonies in de man, rogosa and sharpe (MRS) agar decreased in the first round of treatment at each concentration but increased in subsequent treatments and adaptation processes. The log CFU for curcumin-treated bacteria was 9.2 in the presence of the lowest dose (20 µg/mL) (Fig. 1). The log CFU parameter was marginally raised (up to 100%) following bacteria adaption in the presence of the highest dose of curcumin (150 µg/mL). It is also worth noting that when the curcumin dose was increased to 200 µg/mL, the log CFU of the bacteria decreased significantly and did not adapt to a higher than 150 µg/mL dosage. Therefore, a 150 µg/mL dosage was chosen as the ideal concentration of curcumin to conduct the rest of experiments.

### Resistance to low pH

One of the most distinctive characteristics of all probiotics is their tolerance to low stomach pH, survival rate, and health-promoting properties. In vitro resistance test at low pH was conducted to confirm that curcumin-treated *L. plantarum* can survive in the low pH environment of the stomach. The survival rate of bacteria treated with curcumin, as well as their growth at low pH, did not alter considerably (Table 1).

**Table 1:** Curcumin-treated vs untreated *L. plantarum* survival rates at different time intervals (0, 1, 2, 3 h) following incubation at low pH (pH = 3).

Different groups	Final count (log CFU/ml) of bacteria after incubation at different times				
	0 h	1 h	2 h	3 h	%Viability
Curcumin-treated L. plantarum bacteria	7.13 ± 0.1	7.10 ± 0.2	7.04 ± 0.2	7.04 ± 0.2	98.73
Untreated bacteria	7.13 ± 0.1	7.09 ± 0.2	7.06 ± 0.1	7.05 ± 0.1	98.87

### Evaluation of antibacterial properties of CFS and cur-CFS

**Figure 2:**
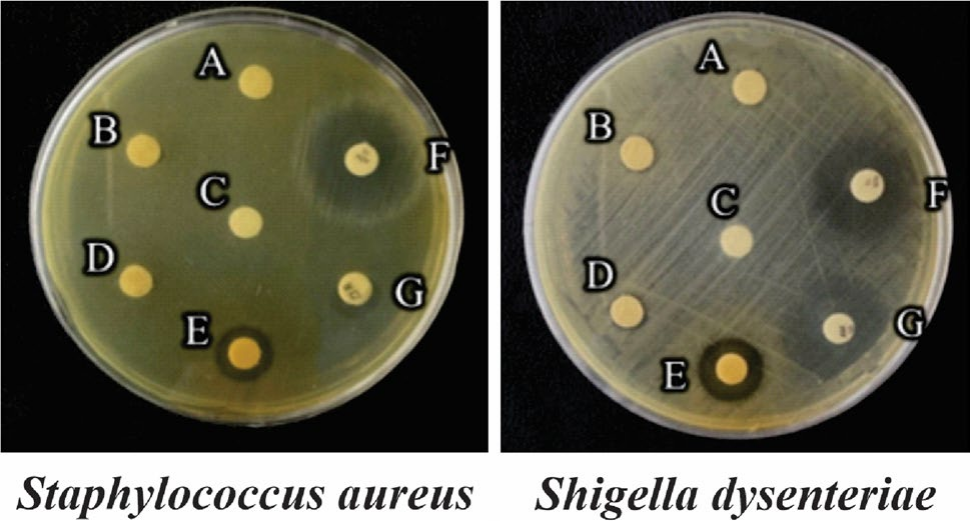
The diameter of the zone of inhibition formed by metabolites derived from untreated and curcumin-treated *L. plantarum* bacteria against *Staphylococcus aureus* and *Shigella dysenteriae*. (**A**) CFS, (**B**) CFS + proteinase k, (**C**) Phosphate buffer as a negative control, (**D**) cur-CFS + proteinase k, (**E**) cur-CFS, (**F**) Azithromycin, (**G**) Ampicillin.

Probiotics are most well-known for their antibacterial capabilities. Probiotics, particularly LAB, produce lactic acid, acetic acid, hydrogen peroxide, and bacteriocins which all have antimicrobial properties. The antibacterial capabilities of CFS and cur-CFS were investigated against various pathogen bacteria to confirm that *L. plantarum* treatment with curcumin did not impact its probiotic feature. Table 2 summarizes the antibacterial activities of CFS and cur-CFS. The results (Fig. 2) indicated that cur-CFS had significantly stronger antibacterial activity against all pathogenic bacteria (*Shigella dysenteriyae*, *Staphylococcus aureus*, *Klebsiella pneumoniae*, *Salmonella typhi*, and *Pseudomonas aeruginosa*) in comparison with CFS.

**Table 2:** Zone of inhibition diameter formed by CFS and cur-CFS against pathogenic bacteria *Shigella dysenteriyae, Staphylococ aureus, Klebsiella pneumoniae, Salmonella typhi,* and *Pseudomonas aeruginosa*.

Strain	Supernatant type	Diameter (mm)
*Staphylococ aureus*	CFS	7.14 ± 0.2
	cur-CFS	13.2 ± 0.3
	CFS + PK	0.00 ± 0.0
	cur-CFS + PK	0.00 ± 0.0
	Positive control (Azithromycin)	20.6 ± 0.3
	Negative control (PBS)	0.00 ± 0.0
*Salmonella typhi*	CFS	8.15 ± 0.3
	cur-CFS	10.9 ± 0.3
	CFS + PK	0.00 ± 0.0
	cur-CFS + PK	0.00 ± 0.0
	Positive control (Azithromycin)	20.5 ± 0.4
	Negative control (PBS)	0.00 ± 0.0
*Pseudomonas aeruginosa*	CFS	8.12 ± 0.4
	cur-CFS	13.2 ± 0.4
	CFS + PK	0.00 ± 0.0
	cur-CFS + PK	0.00 ± 0.0
	Positive control (Azithromycin)	20.4 ± 0.2
	Negative control (PBS)	0.00 ± 0.0
*Shigella dysenteriyae*	CFS	7.40 ± 0.2
	cur-CFS	15.3 ± 0.4
	CFS + PK	0.00 ± 0.0
	cur-CFS + PK	0.00 ± 0.0
	Positive control (Azithromycin)	23.5 ± 0.4
	Negative control (PBS)	0.00 ± 0.0
*Klebsiella pneumonia*	CFS	7.20 ± 0.2
	cur-CFS	12.2 ± 0.3
	CFS + PK	0.00 ± 0.0
	cur-CFS + PK	0.00 ± 0.0
		30.8 ± 0.3
	Positive control (tetracycline)	
	Negative control (PBS)	0.00 ± 0.0

### Characterization of the protein content of CSF

This investigation was done to investigate the nature of CFS and cur-CFS. The pretreatment of cur-CFS with proteinase K, eliminates its antibacterial activity, indicating that the secreted proteins from *L. plantarum* adapted to the highest dose of curcumin are involved in inducing toxicity against pathogenic bacteria (Table 2).

### Cell cytotoxicity

**Figure 3:**
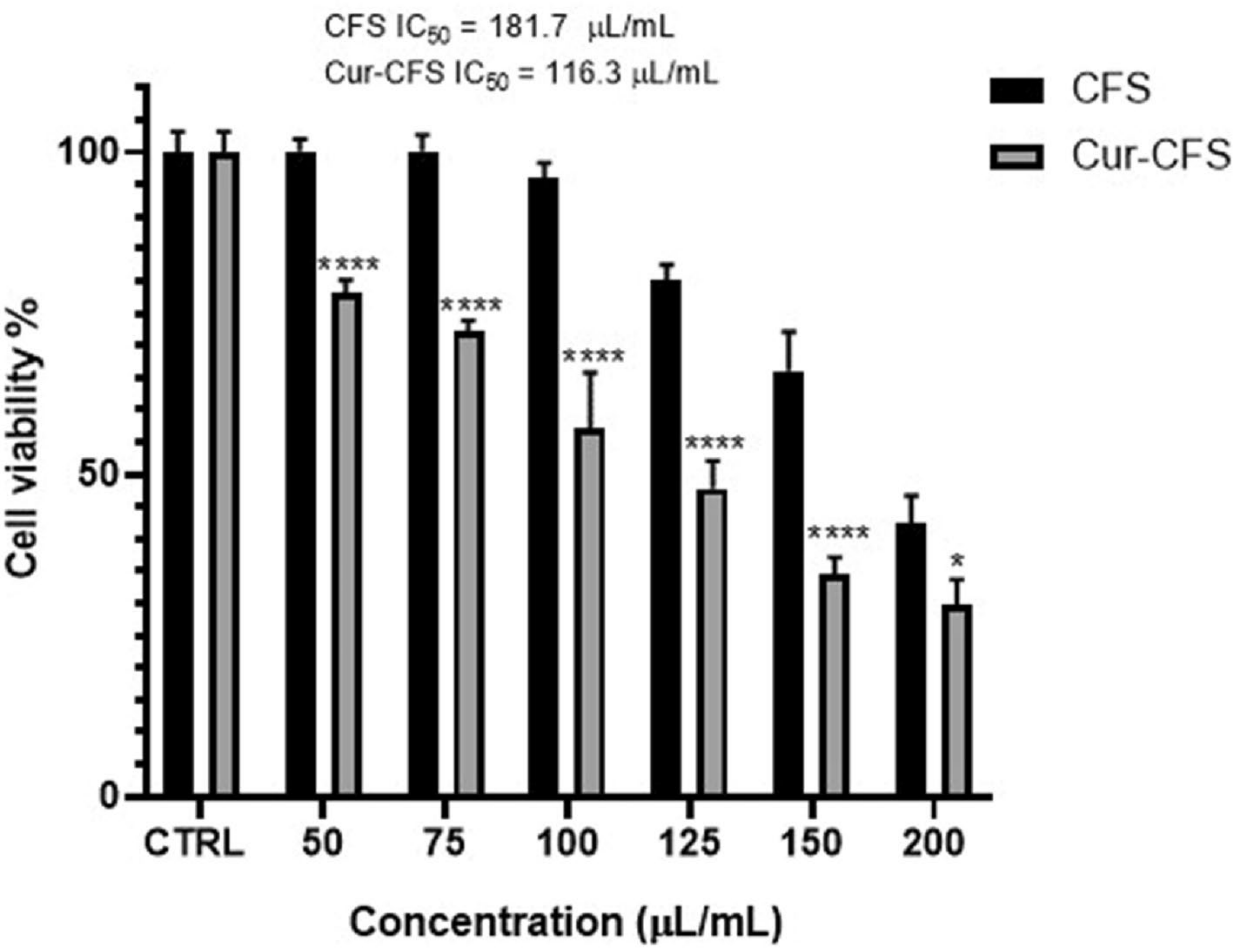
The effect of different concentrations of CFS and cur-CFS on the viability of HT-29 cells after 24 h. (****p ˂ 0.0001) (*p < 0.05).

The MTT assay was used to determine the cytotoxicity effect of CFS and cur-CFS on HT-29 cells^16^. The findings demonstrated that CFS and cur-CFS were capable to induce a cytotoxic effect on HT-29 cells in a dose- and time-dependent manner. A significant decrease in cell viability was observed in 50–150 µL/mL concentrations in the cur-CFS group compared to the CFS group. The decrease in cell viability in 200 µL/mL concentration is less significant (cur-CFS group compared to the CFS group) compared to other concentrations (*P* < 0.05) (Fig. 3). The IC50 value of CFS and cur-CFS were determined as 181.7 μL/ml and 116.3 μL/ml, respectively. Subsequently, the results indicated that cur-CFS could exhibit more cytotoxic effects on (HT-29) in comparison with CFS alone.

### Cur-CFS induced apoptosis in HT-29 cells

**Figure 4:**
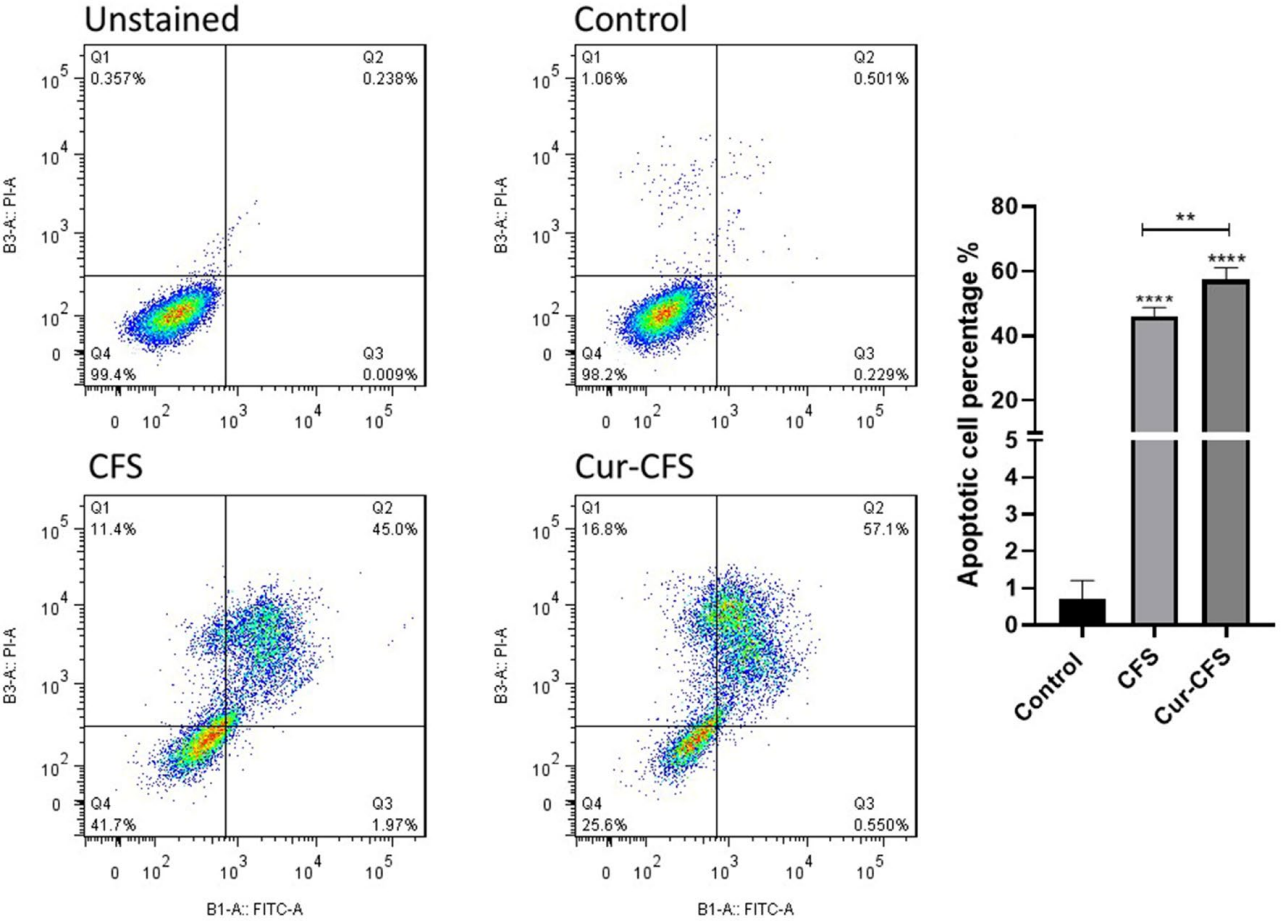
Flow cytometry quantification of apoptosis and necrosis in HT-29 colon cancer cells treated with CFS and cur-CFS in comparison with unstained and positive groups.

To evaluate the effect of CFS and cur-CFS on the apoptosis of HT-29 cells, flow cytometric analysis using a FITC-labeled annexin V kit (ApoFlowEx FITC Kit) was used to identify the early and late phases of apoptosis, as well as the necrosis profiles of the treated cells (Exbio, Czech Republic). The ApoFlowEx FITC Kit is intended for identification of early apoptotic, necrotic and viable cells. Annexin V, a phospholipid-binding protein, binds to phosphatidylserines that translocate to the outer leaflet of the cell membrane during apoptosis. PI-stained cells only with disrupted cell membranes are quantified to identify late apoptotic and necrotic cells^17^. As Fig. 4 shows, the CFS significantly (*p* < 0.0001) increased the rate of apoptosis to 46.97% in HT-29 cells, while this amount was 57.65% in HT-29 cells treated with cur-CFS compared to control cells (*p* < 0.0001). Besides, the apoptosis rate was remarkably higher in cur-CFS-treated cells compared to the CFS-treated cells.

### Cell cycle

**Figure 5:**
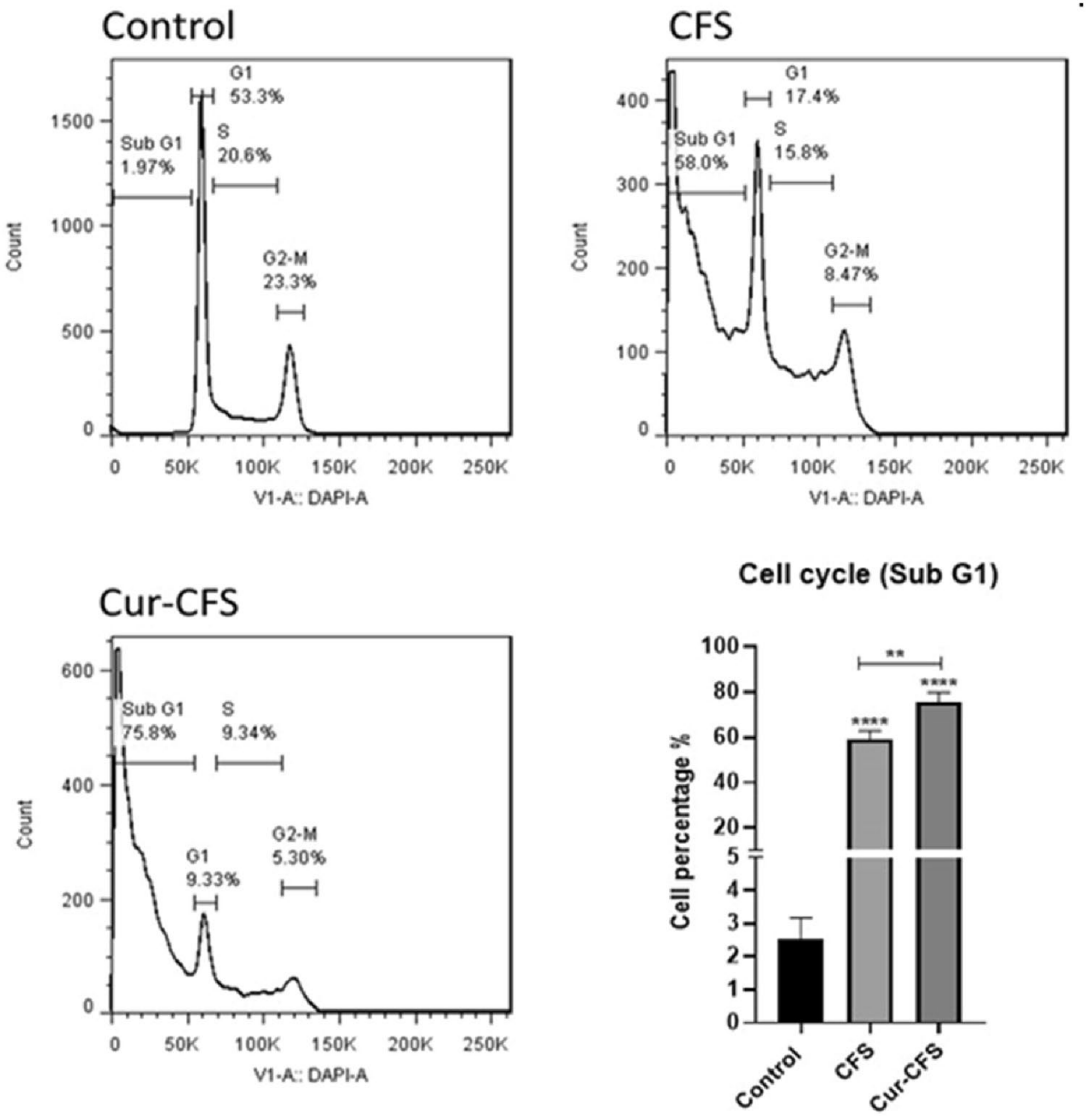
Cell cycle analysis shows the population of cells inters to the Sub-G1 phase in CFS and cur-CFS-treated cells compared to the control group (*p* ˂ 0.0001).

To determine the effect of CFS and cur-CFS, another flow cytometry analysis was performed in HT-29 cells to assess cell cycle arrest. As Fig. 5 shows, 58% of the cells in the CFS-treated cells were arrested in the Sub-G1 stage (cell cycle arrest). In the cur-CFS-treated cells, the arrested cells increased by 75.8%. The statistical analyses results showed a significant difference between the apoptotic cells in the CFS-treated cells and cur-CFS-treated cells (*P* < 0.0001) compared to the control and also a significant difference between these stages in the cur-CFS group compared to CFS (*P* < 0.01).

### DAPI

**Figure 6:**
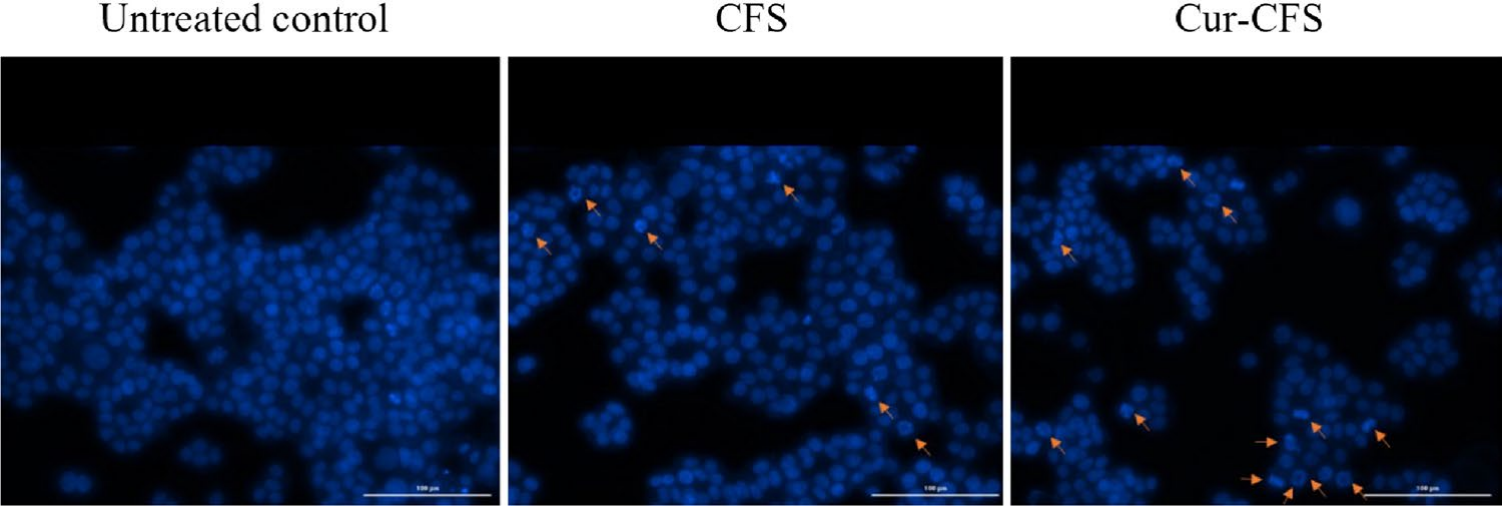
Chromatin changes in HT-29 colon cells after 24 h of treatment with metabolites of intact *L. plantarum* (CFS) and curcumin-treated *L. plantarum* (cur-CFS).

To observe the apoptotic cells in a qualitative manner, the DAPI test was utilized. The nuclei of CFS and cur-CFS-treated HT-29 cells were stained with DAPI to assess the probable chromatin condensation and subsequent DNA fragmentation, which is a hallmark of apoptotic cells. The shape of the nucleus revealed fragmented chromatin in CFS and cur-CFS-treated cells. The modified shape of the nucleus and fragmented DNA in cur-CFS-treated cells was more than in CFS-treated cells. However, the DNA of untreated normal cells remained unaltered (Fig. 6).

### The expression levels of survival/apoptosis-related genes

**Figure 7:**
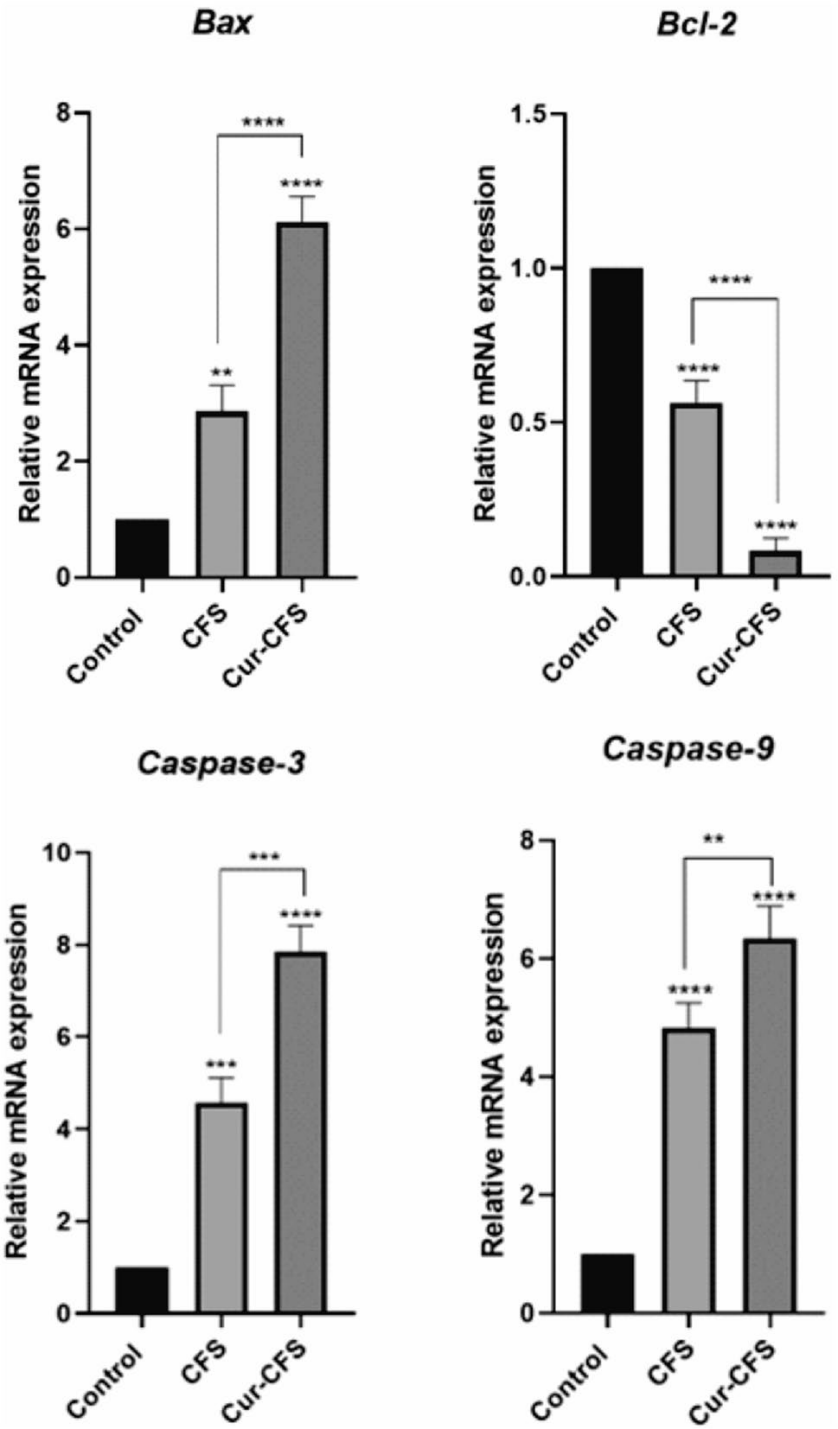
Expression ratios of BAX, Bcl-2, *Caspase-9,* and *Caspase-3* genes in HT-29 colon cancer cells treated with CFS, cur-CFS. The control group was designated as untreated cells (****p ˂ 0.0001).

Quantitative PCR (Q-PCR) was used to measure the amount of gene expression alterations and to further confirm the results of our other tests. The obtained results showed that the Bax gene expression levels in the CFS group (*P* < 0.01) and cur-CFS (*P* < 0.0001) significantly increased compared to the control group. Also, Fig. 7 shows that cur-CFS upregulated the expression of Bax compared to CFS in HT-29 cells (*P* < 0.0001). These results indicate the effect of cur-CFS on the intensification of apoptosis by regulation of this gene. Also, the results showed the downregulation of *Bcl-2* gene in the CFS group (*P* < 0.0001) and cur-CFS (*P* < 0.0001) compared to the control group. Our findings demonstrated that cur-CFS more downregulated the expression of *Bcl-2* gene compared to the CFS group (*P* < 0.0001) as shown in Fig. 7.

Besides, our results illustrated that the *caspase3* gene expression levels in the CFS group (*P* < 0.001) and cur-CFS (*P* < 0.0001) significantly increased compared to the control group. Also, as Fig. 7 shows, its expression was greater in the cur-CFS-treated cells compared to CFS-treated cells (*P* < 0.001). These results indicate the effect of cur-CFS on the intensification of apoptosis by regulation of the *caspase3* gene. Also, comparing the expression of the *caspase9* gene, the results showed that the *caspase9* gene in the CFS-treated cells (*P* < 0.0001) and cur-CFS-treated cells (*P* < 0.0001) compared to the control group had a significant increase in expression which was greater in the cur-CFS-treated cells than in the CFS-treated cells (*P* < 0.01) as shown in Fig. 7.

## Discussion

Cancer is the leading cause of mortality worldwide, making it a serious concern affecting public health on a global scale. The primary cancer treatment approaches are surgery, chemotherapy, immunotherapy, and their combinations^18^. Probiotics have recently sparked considerable interest due to their potential for cancer treatment. Probiotics have been shown to be effective in treating a variety of ailments, including rheumatoid arthritis, psoriasis, autoimmune encephalomyelitis, and gastrointestinal disorders^19–22^. Some of the major health benefits associated with probiotic consumption include the improvement of gastrointestinal microflora, the modulation of the immune system, the reduction of serum cholesterol, the prevention of cancer, the treatment of irritable bowel-associated diarrheas, the effects of antihypertensive medication, and the improvement of lactose metabolism^23^. LAB are the most often utilized probiotics as food supplements in this context. Some LAB strains are thought to boost the immune system, hence preventing colorectal cancer. Oral *Acidophilus Lactobacillus* enhances host immunity by boosting blood IgG and IgM levels as well as mucus IgA levels. Furthermore, LAB or their metabolites can interact directly with tumor cells and inhibit their development^24–28^. The findings of Baldwin et al. indicates that activation of caspase-3 protein was seen following treatment of CRC cells with a mixture of LAB and a chemotherapeutic drug. They suggested that *L. acidophilus* accompanied by LAB strain is able to increase the apoptosis induction^27^. Haghighi et al. investigated the effects of *Streptococcus faecalis*, *Lactobacillus acidophilus*, and *Bifidobacterium bifidum* on antibody response development. They results revealed that probiotics increase the systemic antibody response to certain antigens in chickens^26^. Yurong et al. showed that probiotics enhanced IgA in the intestinal fluid, IgG-forming cells, IgA-forming cells, and IgM-forming cells in the cecal tonsils of chickens. Their results suggested that probiotics enhance the intestinal mucosal immunity of chickens at the early age^28^. Perdigon et al. investigated the antigenic effect of *L. acidophilus* and three other LAB strains on the gut immune system of BALB/c mice. Their results showed that Bcl-2 protein was increased in a dose-dependent manner with all LAB assayed. Their study also demonstrated that LAB strains induce distinct mucosal cytokine profiles^25^. It appears that LAB metabolites play an important therapeutic role and may be linked to microbiota and colorectal cancer. Further investigation revealed that the bacteria possessed a sophisticated mechanism that allowed them to detect nutritional signals and adjust their metabolism accordingly. Evidence suggests that, in addition to heredity, changes in dietary interventions may alter an individual’s microbiomes and metabolomics^29^. Our research group previously has revealed that high dosages of propyl gallate (PG) and tetra butyl hydroquinone (TBHQ) food preservatives might modify the metabolomics of *Lactobacillus rhamnosus* and, as a result, have significant anticancer effects when compared to untreated bacteria^5^. The goal of this study, which was a continuation of previous work, was to examine the effects of curcumin’s natural compound on the anticancer activity of metabolites generated from *L. plantarum* and compare their biological effects with intact bacteria. MTT results demonstrated that CFS and cur-CFS inhibited HT29 cell growth dose-dependently, with a half-maximal inhibitory concentration of 181.7 and 116.3 µL/mL, respectively, at 48 h. DAPI data revealed that cur-CFS-treated cells generated considerably greater chromatin fragmentation within the nucleus than CFS. Furthermore, flow cytometry investigations of apoptosis and cell cycle validated the DAPI staining and MTT assay results, indicating that cur-CFS-treated cells had a greater incidence of apoptosis (about 57.65%) than CFS-treated cells (approximately 47 percent). cur-CFS and CFS increased the expression of the Caspase 9,3 and BAX genes while decreasing the expression of the BCL-2 gene, as verified by qPCR.

Dehghani et al. investigated the cytotoxicity, anti-proliferative effects, and apoptotic properties of the supernatant of probiotic *Lactobacillus rhamnosus* on the HT-29 cell line. Their findings were in line with our results. Their results showed that the supernatant of *L. rhamnosus* inhibited the development of HT-29 cancer cells in a dose- and time-dependent manner. Moreover, they affirmed the apoptotic cell death by flow cytometry and qPCR. We have also observed similar results after pre-treatment of the *L. plantarum* with curcumin^30^.

## Materials and methods

### Materials

The human colorectal adenocarcinoma cell line (HT-29) was purchased from the Pasteur Institute. All of the strains of *Lactobacillus plantarum* PTCC 1058, *Shigella dysenteriyae*, *Staphylococ aureus*, *Klebsiella pneumoniae*, and *Salmonella typhi* were bought from the Iranian Research Organization for Science and Technology, Ministry of Science, Technology, and Research (Tehran, Iran). RPMI 1640 medium, trypsin, FBS, streptomycin, and penicillin to were supplied by Invitrogen (Waltham, MA, USA). Pipettes, tissue culture flasks, and 96-well and 6-well plates were purchased from SPL Life Sciences (Gyeonggi-do, South Korea). Proteinase K, trypan-blue, MTT powder, diethylpyrocarbonate (DEPC), Triton-X100, and sterile disks were obtained from Sigma Aldrich (St. Louis, MI, USA). DMSO, DAPI, curcumin, de Man, Rogosa, and Sharpe (MRS) agar broth and MRS agar media were provided by Merck (Darmstadt, Germany). RNA isolation kit (RNX plus total) was bought from Genall. The Reverta-L reagent kit was purchased from InterLabService (Moscow, Russia). Power SYBR Green PCR master mix was obtained from Applied Biosystems (Foster City, CA, USA). Propidium iodide (PI) and annexin V-fluorescein isothiocyanate (FITC) kits were purchased from EBioscience (Waltham, MA, USA). All extra materials, unless otherwise mentioned, were purchased from Merck (Darmstadt, Germany) or Fermentas Life Science (Waltham, MA, USA).

### Preparation of cell-free supernatants (CFS)

*L. plantarum* was cultured in a customized MRS broth medium (37 °C) overnight to achieve an optical density (OD) of 0.7–0.8 at 600 nm (ELx 800 spectrophotometer; Biotek, San Francisco, CA, USA). MRS agar is developed primarily for the cultivation of lactobacilli from various sources with the intention of producing a defined medium as a substitute for tomato juice agar. Plate counting on MRS agar found the OD (0.8) yield to be 10^9^ colony-forming units (CFU)/mL. The bacterial density corresponded to the number of bacteria found in the large intestine (i.e., about 10^9^–10^11^ CFU/mL). In brief, cultured bacteria in the presence of curcumin displayed resistance to the greatest dosages of curcumin in incremental concentrations (20–40–60–80–100–150–200 µg/mL) during repeated cultures. The bacteria were cultivated in each concentration of curcumin for 48 h until the number of bacterial colonies cultured in MRS broth reached 10^9^ CFU/mL. The bacteria that were resistant to the highest dose of curcumin (200 µg/mL) and the untreated bacteria were centrifuged (400×*g*, 5 min) and their supernatant and bacterium sediment were collected. The supernatants of curcumin-treated bacteria and intact bacteria were adjusted to a pH of 7.4 and filtered through a 0.2 filter to yield cell-free supernatants of intact bacteria (CFS) and bacteria resistant to curcumin (cur-CFS). The bacterium sediments were washed twice with PBS (pH 7.4) and tested for tolerance to low pH. To undertake cellular studies, both curcumin-treated and untreated bacteria were centrifuged 24 h before treatment and transferred to an RPMI medium. After 24 h, the supernatants were centrifuged to collect the CFS and cur-CFS, and their pH was adjusted to 7.4 and filtered using a 0.2 filter.

### Resistance to low pH environments

To test the resistance of curcumin-treated bacteria to the stomach acidic environment, a technique published by was modified and applied^31^. The bacteria that were resistant to the highest dose of curcumin were separated and washed with PBS (pH 7.2). The washed bacteria were immersed in phosphate buffer solution (PBS) (0.1 M NaH_2_PO_4_ and 0.1 M Na_2_HPO_4_) pH 3.0 for 0, 1, 2, and 3 h. The bacteria's resistance rate was evaluated on MRS agar using the pour plate technique, and the survival of treated and untreated cells was compared. Using equation (I), the survival rate of bacteria resistant to the greatest dosage of curcumin was calculated and compared to the intact bacteria:

1 $${\text{Survival}}\,{\text{rate}}\% = {\text{log}}\,{\text{CFUN}}_{{1}} /{\text{log CFUN}}_{0} \times {1}00$$

where N_1_ represents the total viable counts of isolated bacteria in the MRS agar at low pH; and N_0_ is the total viable counts of bacteria before cultivation in the acidic media.

### Zone of inhibition assay

A disk diffusion test was performed to assess CFS's antibacterial capabilities against clinically pathogenic bacteria. (Gram-negative bacteria *Shigella dysenteriyae* were used and the gram-positive bacteria included*: Staphylococcus aureus, Klebsiella pneumoniae, Salmonella typhi,* and *Pseudomonas aeruginosa*)^31^. To summarize, all of the bacterial strains were cultivated in nutritional broths. A sterile disk (5 mm) containing of CFS and cur-CSF (10 µl/mL), and appropriate antibiotics were placed on the culture plate and allowed to diffuse into the agar for 2 h at 24 °C. The plates were then incubated for 24 h at 37 °C before being tested to determine the width of the inhibitory zone. The inhibition zones surrounding the disks were measured with a Mitutoyo sliding caliper, and the findings were represented as resistance (0 mm), moderate resistance (0–4 mm), moderate susceptibility (4–8 mm), susceptibility (8–12 mm), and extra susceptibility (> 12 mm) in comparison to standards antibiotic^32^. A sterile disk with PBS served as the negative control (pH 7.2). Azithromycin (30 µg) and Ampicillin (30 µg) were used as positive controls for *Pseudomonas aeruginosa, Shigella dysenteriyae, Salmonella typhi, and Staphylococcus aureus* (Bio-Rad, Hercules, CA, USA). Tetracycline and cefazolin (30 µg) were employed as positive controls for *Klebsiella pneumoniae.*

### Characterization of the protein content of CSF

The prepared CSFs (200 µl/mL) were treated for 1 h at 37 °C with proteinase K (1 µl/mL) to assess the protein components of CFS. The antibacterial and cytotoxicity activity of protein-free CSFs were compared to intact CFSs after being inactivated by proteinase K for 20 min at 70 °C^33^.

### Cell culture

HT-29 human colorectal adenocarcinoma cells were grown at 37 °C in RPMI 1640 medium supplemented with 10% FBS and antibiotics 100 U/mL penicillin and 100 µL/mL streptomycin in a water-saturated atmosphere containing 5% CO_2_.

### Cell viability assay

The MTT assay was used to analyze the cytotoxic effects of CFSs on HT-29 cells in 96-well plates^34,35^. Cells were seeded at a density of 2.0 × 10^4^ cells/well for 24 h. The cells were subsequently incubated at 37 °C for 24 h with different dosages of CFSs dissolved in RPMI. Following the incubation durations, the media were removed and 200 µL of MTT reagent (0.5 mg/mL) was added to each well before incubating for 4 h at 37 °C in the humidified CO_2_ incubator. After 4 h of incubation, the formazan crystals formed by the oxidation of the MTT dye in living cells were dissolved in DMSO and the UV absorbance at 570 nm was determined using a Tecan ELISA plate reader (Männedorf, Switzerland).

### Annexin V/FITC apoptosis/necrosis detection

The ApoFlowEx FITC Kit was used to identify the early and late phases of apoptosis, as well as the necrosis profiles of the treated cells (Exbio, Czech Republic). Briefly, treated cells were rinsed (× 3) with PBS, trypsinized, and then washed (× 3) with 500 µL of binding buffer (BB) [Tris–HCl (1 m, pH 8.0), EDTA (500 mm), and NaCl (5 m)]^36,37^. After draining the supernatant, the cells were resuspended in 100 µL of BB and 5 µL of annexin V-FITC. After 15 min of incubation at 24 ± 2 °C in dim light, the cells were washed once more with BB. Following supernatant removal, 100 μL of the BB and 5 μL of PI staining solution were added and incubated for additional 5 min at 24 ± 2 °C in the dark. The cells were examined using a BD FACSCaliburTM flow cytometer (FACSQuant; Milteny, Germany) equipped with FITC (green) emission filters of 515–545 nm and PI emission filters of 600 nm (red). Each experiment recorded a total of 10,000 occurrences.

### DAPI staining

DAPI staining was used to determine the extent of DNA breakage (condensed and fragmented DNA) generated by various CFSs in the HT-29 cell line using an Olympus IX81 fluorescent microscope (Hamburg, Germany)^5^. To do this, the cells were seeded at a density of 5.0 × 10^5^ cells/well in 6-well plates with 22 cm^2^ coverslips. After 24 h, the cells were treated with 150 µL/mL CFSs (concentrations corresponding to the 24-h IC50, respectively). As a positive control, DMSO (5% v/v) was utilized. After 24 h of incubation, the cells were rinsed with PBS and fixed for 1 h at 24 ± 2 °C with 4% paraformaldehyde. After washing with PBS, the cells were permeabilized with Triton X- 100 (0.1% v/v) and stained with DAPI (200 ng/mL). Finally, the images of treated cells were taken using a fluorescent microscope (excitation at 350–360 nm and emission at 455–465 nm).

#### Cell cycle analysis

Cell cycle analysis was performed to investigate the apoptotic properties of CFS and cur-CFS. In brief, the cells were removed from the 6-well plates with Trypsin/EDTA solution and rinsed twice with cold PBS. Then, 1 mL of 75% ethanol was used for each group of the cells in order to fixation and incubated at − 20 °C overnight. After 24 incubations, the cells were then washed with PBS and 5 μL of RNase A was added to each group and incubated for 30 min at 37 °C. Afterward, 20 μL PI alongside Triton X-100 (0.1% v/v) was added to the cells. The cell cycle arrest was detected with flow cytometry and analyzed by FlowJo software as well.

#### Real-time reverse transcription-polymerase chain reaction (RT-PCR)

The effect of various CFSs on expression levels of Bax, *caspase9,* and *caspase3* (pro-apoptotic gene), and *Bcl-2* (anti-apoptotic gene) was evaluated using quantitative real-time PCR analysis. The expression level of GAPDH was also investigated as the housekeeping gene. To evaluate the effect of CFS on the expression of specific genes, HT-29 cells were cultured in 6-well plates for 24 h and treated with various CFSs at corresponding IC50 concentrations. The cells were lysed for RNA extraction after 24 h of incubation at 37 °C^38^. A Real-time PCR reaction was performed in a Bio-Rad device. For each gene, reverse and forward primers (Table 3) were designed by oligo 7.56 software and provided by Takapozist (Tehran, Iran).

**Table 3:** Sequences and properties of primers used in RT-PCR.

Gene name	Primers	Primer sequence (5′ → 3′)	Length	Gen bank accession number
BAX	Forward	GACTCCCCCCGAGAGGTCTT	121	NM_004324
	Reverse	ACAGGGCCTTGAGCACCAGTT		
BCL-2	Forward	CTGTGGATGACTGAGTACCTG	127	NM_000633
	Reverse	GAGACAGCCAGGAGAAATCA		
Caspase-3	Forward	CAAACCTCAGGGAAACATTCAG	137	NM_004346
	Reverse	CACACAAACAAAACTGCTCC		
Caspase-9	Forward	CTGTCTACGGCACAGATGGAT	177	NM_001229
	Reverse	GGGACTCGTCTTCAGGGGAA		
GAPDH	Forward	CAAGATCATCAGCAATGCCT	166	NM_002046
	Reverse	GCCATCACGCCACAGTTTCC		

The qPCR reaction was performed using SYBR® Green master mixes. This reaction was performed at a volume of 20 μL/well, each containing 10 μL of master mix, 1 μL of cDNA, and 0.5 μL of each of the forward and reverse primers, which reached a volume of 20 μL with diethylpyrocarbonate (DEPC) water. Thermal cycling conditions were as follows: 10 min at 94 °C, 40 cycles at 95 °C for 15 s, 30 s at 60–62 °C (depending on the gene utilized), and 25 s at 72 °C. The Pfaffl technique was used to examine the data, and the cycle threshold (CT) values were standardized to the rate of β-actin expression. Each reaction was performed in triplicate, and each experiment included a negative control.

#### Statistical analysis

At least three independent tests were done on each set of data collected throughout this investigation. The mean and standard deviation of continuous variables were used to express them (SD). The analysis of variance (ANOVA) followed by Dunnett’s test was performed to determine the significant differences between the groups. GraphPad Prism version 6.01 was used to conduct all statistical analyses. *P* ˂ 0.05 was considered statistically significant.

## Conclusion

Probiotic bacteria have been shown to have anticancer properties against various types of cancers, including colorectal cancer. According to research, probiotic bacteria have a specific mechanism that allows them to receive nutritional signals and adjust their metabolism accordingly based on their prebiotics. Previous research has suggested that there is a relationship between nutrition with probiotic biological activities. Curcumin is a food additive that is popular in many cuisines due to its spicy flavor and vibrant color. Long-term curcumin use may affect the biological characteristics of probiotic bacteria in individuals, particularly their anticancer properties. Therefore, in this investigation curcumin was used to assess its properties on the antiproliferative properties of the probiotic *L. plantarum* strain. In this context, *L. plantarum* bacteria were treated with curcumin and their probiotic features were evaluated in terms of their low pH tolerance potency and antibacterial properties. Furthermore, the antiproliferative activity of the CFS of curcumin-treated *L. plantarum* was compared to that of intact *L. plantarum*. Curcumin-treated *L. plantarum* caused more apoptosis against HT-29 colon cancer cells than intact *L. plantarum* , as demonstrated by cytotoxicity and genotoxicity studies. The findings in this study suggest that the metabolomics of curcumin-treated probiotics may either directly induce enhanced toxicity against cancer cells or may interact with curcumin indirectly, amplifying their anticancer effects.

## Data Availability

The datasets used and/or analyzed during the current study are available from the corresponding author upon reasonable request.

## References

[1] Marquardt JU, Galle PR, Teufel A (2012). Molecular diagnosis and therapy of hepatocellular carcinoma (HCC): An emerging field for advanced technologies. J. Hepatol..

[2] Basch CH, Hillyer GC, Jacques ET (2022). News coverage of colorectal cancer on google news: Descriptive study. JMIR Cancer.

[3] Carpenter S (2012). That gut feeling. Monit. Psychol..

[4] Kaur IP, Chopra K, Saini A (2002). Probiotics: Potential pharmaceutical applications. Eur. J. Pharm. Sci..

[5] Salmanzadeh R (2018). Propyl gallate (PG) and tert-butylhydroquinone (TBHQ) may alter the potential anti-cancer behavior of probiotics. Food Biosci..

[6] De Wever O, Mareel M (2003). Role of tissue stroma in cancer cell invasion. J. Pathol. A J. Pathol. Soc. Great Br. Irel..

[7] Rafter J (2004). The effects of probiotics on colon cancer development. Nutr. Res. Rev..

[8] Dunne C, Murphy L, Flynn S (1999). Probiotics: from myth to reality. Demonstration of functionality in animal models of disease and in human clinical trials. Antonie Van Leeuwenhoek.

[9] Farag MA (2020). Metabolomics reveals impact of seven functional foods on metabolic pathways in a gut microbiota model. J. Adv. Res..

[10] Chainani-Wu N (2003). Safety and anti-inflammatory activity of curcumin: A component of tumeric (Curcuma longa). J. Altern. Complement. Med..

[11] Abd El-Hack ME (2021). Curcumin, the active substance of turmeric: Its effects on health and ways to improve its bioavailability. J. Sci. Food Agricult..

[12] López-Lázaro M (2008). Anticancer and carcinogenic properties of curcumin: Considerations for its clinical development as a cancer chemopreventive and chemotherapeutic agent. Mol. Nutr. Food Res..

[13] Mohammadian Haftcheshmeh S (2020). Modulatory effects of curcumin on the atherogenic activities of inflammatory monocytes: Evidence from in vitro and animal models of human atherosclerosis. BioFactors.

[14] Stanić Z (2017). Curcumin, a Compound from Natural Sources, a True Scientific Challenge – A Review. Plant Foods Hum. Nutr..

[15] Spyropoulos BG, Misiakos EP, Fotiadis C, Stoidis CN (2011). Antioxidant properties of probiotics and their protective effects in the pathogenesis of radiation-induced enteritis and colitis. Dig. Dis. Sci..

[16] Derakhshankhah H (2021). A bio-inspired gelatin-based pH- and thermal-sensitive magnetic hydrogel for in vitro chemo/hyperthermia treatment of breast cancer cells. J. Appl. Polym. Sci..

[17] Bahadori MB (2019). Triterpenoid corosolic acid attenuates HIF-1 stabilization upon cobalt (II) chloride-induced hypoxia in A549 human lung epithelial cancer cells. Fitoterapia.

[18] Giaquinto AN (2022). Cancer statistics for African American/black people 2022. CA A Cancer J. Clin..

[19] Sarowska J, Choroszy-Król I, Regulska-Ilow B, Frej-Madrzak M, Jama-Kmiecik A (2013). The therapeutic effect of probiotic bacteria on gastrointestinal diseases. Adv Clin Exp Med.

[20] Mohammed AT (2017). The therapeutic effect of probiotics on rheumatoid arthritis: A systematic review and meta-analysis of randomized control trials. Clin. Rheumatol..

[21] Alesa DI (2019). The role of gut microbiome in the pathogenesis of psoriasis and the therapeutic effects of probiotics. J. Fam. Med. Primary Care.

[22] Lavasani S (2010). A novel probiotic mixture exerts a therapeutic effect on experimental autoimmune encephalomyelitis mediated by IL-10 producing regulatory T cells. PLoS ONE.

[23] Shi LH, Balakrishnan K, Thiagarajah K, Ismail NIM, Yin OS (2016). Beneficial properties of probiotics. Trop. Life Sci. Res..

[24] Tejada-Simon M, Lee J, Ustunol Z, Pestka J (1999). Ingestion of yogurt containing *Lactobacillus acidophilus* and Bifidobacterium to potentiate immunoglobulin A responses to cholera toxin in mice. J. Dairy Sci..

[25] Perdigon G, Maldonado Galdeano C, Valdez J, Medici M (2002). Interaction of lactic acid bacteria with the gut immune system. Eur. J. Clin. Nutr..

[26] Haghighi HR (2005). Modulation of antibody-mediated immune response by probiotics in chickens. Clin. Vaccine Immunol..

[27] Baldwin C (2010). Probiotic *Lactobacillus acidophilus* and L. casei mix sensitize colorectal tumoral cells to 5-fluorouracil-induced apoptosis. Nutr. Cancer.

[28] Yurong Y, Ruiping S, ShiMin Z, Yibao J (2005). Effect of probiotics on intestinal mucosal immunity and ultrastructure of cecal tonsils of chickens. Arch. Anim. Nutr..

[29] Pryor R (2019). Host-microbe-drug-nutrient screen identifies bacterial effectors of metformin therapy. Cell.

[30] Dehghani N, Tafvizi F, Jafari P (2021). Cell cycle arrest and anti-cancer potential of probiotic *Lactobacillus rhamnosus* against HT-29 cancer cells. Bioimpacts.

[31] Nami A, Liang J, Dijkhuizen F, Demetriades GD (2014). Modular multilevel converters for HVDC applications: Review on converter cells and functionalities. IEEE Trans. Power Electron..

[32] Reller LB, Weinstein M, Jorgensen JH, Ferraro MJ (2009). Antimicrobial susceptibility testing: A review of general principles and contemporary practices. Clin. Infect. Dis..

[33] Ma W-X, Huang T, Zhang Y (2010). A multiple exp-function method for nonlinear differential equations and its application. Phys. Scr..

[34] Vandghanooni S, Eskandani M, Barar J, Omidi Y (2020). Antisense LNA-loaded nanoparticles of star-shaped glucose-core PCL-PEG copolymer for enhanced inhibition of oncomiR-214 and nucleolin-mediated therapy of cisplatin-resistant ovarian cancer cells. Int. J. Pharm..

[35] Jaymand M (2016). Development of novel electrically conductive scaffold based on hyperbranched polyester and polythiophene for tissue engineering applications. J. Biomed. Mater. Res. Part A.

[36] Hamishehkar H (2018). Preparation, characterization and anti-proliferative effects of sclareol-loaded solid lipid nanoparticles on A549 human lung epithelial cancer cells. J. Drug Deliv. Sci. Technol..

[37] Eskandani M (2022). Protective effect of L-carnitine-loaded solid lipid nanoparticles against H_2_O_2_-induced genotoxicity and apoptosis. Colloids Surf. B Biointerfaces.

[38] Vandghanooni S (2011). Survivin-deltaEx3: A novel biomarker for diagnosis of papillary thyroid carcinoma. J. Cancer Res. Ther..

